# Benzotriazolium 4-methyl­benzene­sulfonate

**DOI:** 10.1107/S1600536814003857

**Published:** 2014-02-22

**Authors:** A. Thirunavukkarasu, A. Silambarasan, R. Mohan Kumar, P. R. Umarani, G. Chakkaravarthi

**Affiliations:** a Department of Physics, Presidency College, Chennai 600 005, India; b Kunthavai Naacchiyaar Govt. Arts College (W), Thanjavur 613 007, India; c Department of Physics, CPCL Polytechnic College, Chennai 600 068, India

## Abstract

In the title molecular salt, C_6_H_6_N_3_^+^·C_7_H_7_O_3_S^−^, the components are linked by N—H⋯O hydrogen bonds into zigzag chains along [100]. These chains are further connected by weak C—H⋯O, C—H⋯π and π–π (centroid-to-centroid distances = 3.510, 3.701 and 3.754 Å) inter­actions into a three-dimensional network.

## Related literature   

For biological activities of benzotriazole derivates, see: Dubey *et al.* (2011 ▶); Gaikwad *et al.* (2012 ▶). For related structures, see: Sudhahar *et al.* (2013 ▶); Yang *et al.* (2010 ▶). 
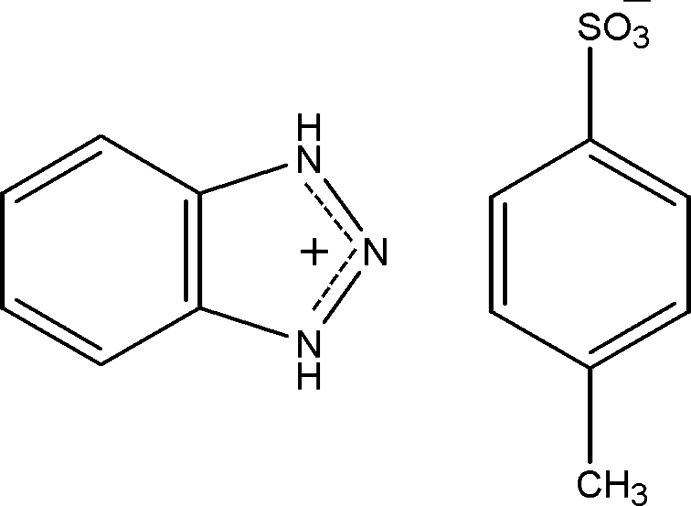


## Experimental   

### 

#### Crystal data  

C_6_H_6_N_3_^+^·C_7_H_7_O_3_S^−^
*M* *_r_* = 291.32
Orthorhombic, 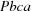

*a* = 12.2330 (5) Å
*b* = 13.4144 (6) Å
*c* = 16.3320 (9) Å
*V* = 2680.1 (2) Å^3^
*Z* = 8
Mo *K*α radiation
μ = 0.25 mm^−1^
*T* = 295 K
0.26 × 0.24 × 0.20 mm

#### Data collection  

Bruker Kappa APEXII CCD diffractometer
Absorption correction: multi-scan (*SADABS*; Sheldrick, 1996 ▶) *T*_min_ = 0.937, *T*_max_ = 0.951
45507 measured reflections
3889 independent reflections
2766 reflections with *I* > 2σ(*I*)
*R*_int_ = 0.037

#### Refinement  

*R*[*F* ^2^ > 2σ(*F* ^2^)] = 0.040
*wR*(*F* ^2^) = 0.108
*S* = 1.08
3889 reflections
191 parameters
2 restraints
H atoms treated by a mixture of independent and constrained refinement
Δρ_max_ = 0.26 e Å^−3^
Δρ_min_ = −0.35 e Å^−3^

### 

Data collection: *APEX2* (Bruker, 2004 ▶); cell refinement: *SAINT* (Bruker, 2004 ▶); data reduction: *SAINT*; program(s) used to solve structure: *SHELXS97* (Sheldrick, 2008 ▶); program(s) used to refine structure: *SHELXL97* (Sheldrick, 2008 ▶); molecular graphics: *PLATON* (Spek, 2009 ▶); software used to prepare material for publication: *SHELXL97*.

## Supplementary Material

Crystal structure: contains datablock(s) global, I. DOI: 10.1107/S1600536814003857/bt6963sup1.cif

Structure factors: contains datablock(s) I. DOI: 10.1107/S1600536814003857/bt6963Isup2.hkl

Supporting information file. DOI: 10.1107/S1600536814003857/bt6963Isup3.cml

CCDC reference:

Additional supporting information:  crystallographic information; 3D view; checkCIF report

## Figures and Tables

**Table 1:** Hydrogen-bond geometry (Å, °) *Cg*1 is the centroid of the C1–C6 ring.

*D*—H⋯*A*	*D*—H	H⋯*A*	*D*⋯*A*	*D*—H⋯*A*
N2—H2*A*⋯O2^i^	0.87 (1)	1.82 (1)	2.661 (2)	161 (2)
N3—H3*A*⋯O3^ii^	0.87 (1)	1.77 (1)	2.6326 (19)	176 (2)
C3—H3⋯O2^iii^	0.93	2.48	3.359 (2)	158
C12—H12⋯*Cg*1^iv^	0.93	2.66	3.500 (2)	150

Symmetry codes: (i) 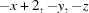; (ii) 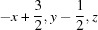; (iii) 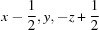; (iv) 
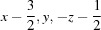
.

**Fig. 1.**
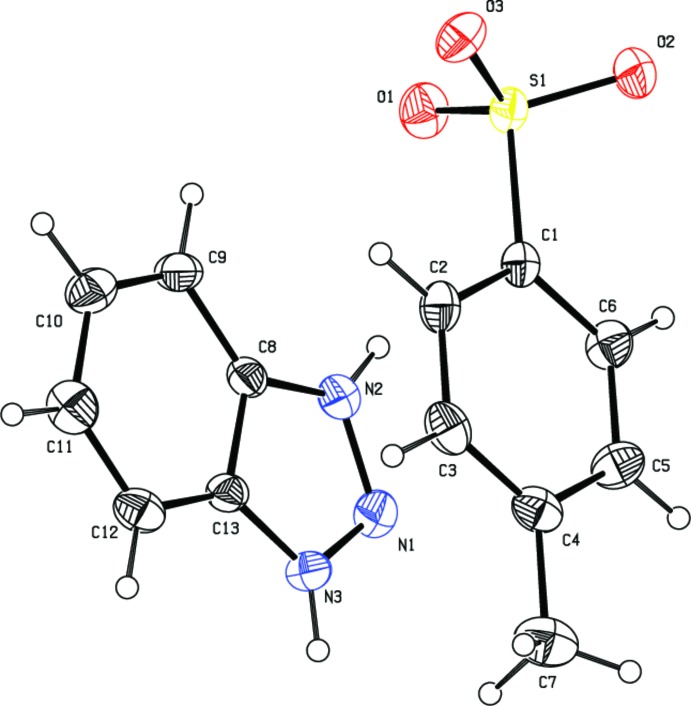
The molecular structure of the title compound, with atom labels and 30% probability displacement ellipsoids for non-H atoms.

**Fig. 2.**
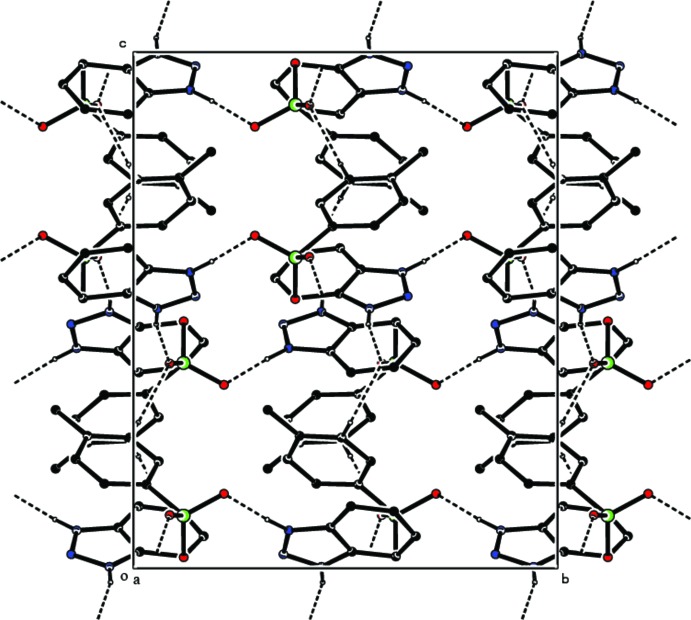
The packing of the title compound, viewed down a axis. Intermolecular hydrogen bonds are shown as dashed lines. H atoms not involved in hydrogen bonding have been omitted.

## supplementary crystallographic information

### 1. Comment

Benzotriazole derivates exhibit antitubercular and antimicrobial (Dubey *et al.*, 2011; Gaikwad *et al.*, 2012) bio-activities. We herewith report the crystal structure of the title compound (Fig. 1). In the title compound, the geometric parameters are comparable with reported structures (Sudhahar *et al.*, 2013; Yang *et al.*, 2010).

The crystal structure exhibits intermolecular N-H···O, C-H···O, C-H···π (Table 1; Fig. 2) and π···π interactions [Cg1···Cg2^i^: 3.510Å; Cg2···Cg3^ii^: 3.754Å; Cg3···Cg3^ii^: 3.701Å; symmetry operators: (i) x,y,z; (ii) 1-x,-y,-z; Cg1, Cg2 and Cg3 are the centroids of the rings (C1-C6), (N1/N2/C8/C13/N3) and (C8-C13), respectively].

### 2. Experimental

Benzotriazole (C_6_H_5_N_3_, 1.1913 g) and p-toluenesulfonic acid (CH_3_C_6_H_4_S0_3_H, 1.90 g) were taken in an equimolar ratio and dissolved in methanol and water. The solution was allowed for slow evaporation at room temperature. The crystals were collected after a the period of 45 days.

### 3. Refinement

All H atoms were located in a difference Fourier map. N-bound H atoms were refined with an N-H distance restraint of 0.86 (1)Å. The C-bound H atoms were positioned geometrically and refined using a riding model with C—H = 0.93 and *U*_iso_(H) = 1.2*U*_eq_(C) for CH and C—H = 0.96 Å and *U*_iso_(H) = 1.5*U*_eq_(C) for CH_3_.

### Crystal data

**Table:**

C_6_H_6_N_3_^+^·C_7_H_7_O_3_S^−^	*F*(000) = 1216
*M**_r_* = 291.32	*D*_x_ = 1.444 Mg m^−^^3^
Orthorhombic, *P**b**c**a*	Mo *K*α radiation, λ = 0.71073 Å
Hall symbol: -P 2ac 2ab	Cell parameters from 9880 reflections
*a* = 12.2330 (5) Å	θ = 2.5–29.4°
*b* = 13.4144 (6) Å	µ = 0.25 mm^−^^1^
*c* = 16.3320 (9) Å	*T* = 295 K
*V* = 2680.1 (2) Å^3^	Block, colourless
*Z* = 8	0.26 × 0.24 × 0.20 mm

### Data collection

**Table:**

Bruker Kappa APEXII CCD diffractometer	3889 independent reflections
Radiation source: fine-focus sealed tube	2766 reflections with *I* > 2σ(*I*)
Graphite monochromator	*R*_int_ = 0.037
ω and φ scan	θ_max_ = 30.5°, θ_min_ = 2.5°
Absorption correction: multi-scan (*SADABS*; Sheldrick, 1996)	*h* = −17→17
*T*_min_ = 0.937, *T*_max_ = 0.951	*k* = −18→18
45507 measured reflections	*l* = −22→23

### Refinement

**Table:**

Refinement on *F*^2^	Secondary atom site location: difference Fourier map
Least-squares matrix: full	Hydrogen site location: inferred from neighbouring sites
*R*[*F*^2^ > 2σ(*F*^2^)] = 0.040	H atoms treated by a mixture of independent and constrained refinement
*wR*(*F*^2^) = 0.108	*w* = 1/[σ^2^(*F*_o_^2^) + (0.0359*P*)^2^ + 1.3937*P*] where *P* = (*F*_o_^2^ + 2*F*_c_^2^)/3
*S* = 1.08	(Δ/σ)_max_ < 0.001
3889 reflections	Δρ_max_ = 0.26 e Å^−^^3^
191 parameters	Δρ_min_ = −0.35 e Å^−^^3^
2 restraints	Extinction correction: *SHELXL97* (Sheldrick, 2008), Fc^*^=kFc[1+0.001xFc^2^λ^3^/sin(2θ)]^-1/4^
Primary atom site location: structure-invariant direct methods	Extinction coefficient: 0.0071 (5)

### Special details

**Table:**

Geometry. All esds (except the esd in the dihedral angle between two l.s. planes) are estimated using the full covariance matrix. The cell esds are taken into account individually in the estimation of esds in distances, angles and torsion angles; correlations between esds in cell parameters are only used when they are defined by crystal symmetry. An approximate (isotropic) treatment of cell esds is used for estimating esds involving l.s. planes.
Refinement. Refinement of F^2^ against ALL reflections. The weighted R-factor wR and goodness of fit S are based on F^2^, conventional R-factors R are based on F, with F set to zero for negative F^2^. The threshold expression of F^2^ > 2sigma(F^2^) is used only for calculating R-factors(gt) etc. and is not relevant to the choice of reflections for refinement. R-factors based on F^2^ are statistically about twice as large as those based on F, and R- factors based on ALL data will be even larger.

### Fractional atomic coordinates and isotropic or equivalent isotropic displacement parameters (Å^2^)

**Table:**

	*x*	*y*	*z*	*U*_iso_*/*U*_eq_	
C1	0.90878 (12)	0.03041 (12)	0.16244 (9)	0.0333 (3)	
C2	0.81836 (14)	0.05952 (13)	0.20733 (11)	0.0418 (4)	
H2	0.7961	0.1258	0.2066	0.050*	
C3	0.76102 (15)	−0.00918 (15)	0.25324 (11)	0.0460 (4)	
H3	0.6998	0.0114	0.2824	0.055*	
C4	0.79293 (15)	−0.10826 (14)	0.25669 (11)	0.0432 (4)	
C5	0.88410 (16)	−0.13637 (13)	0.21152 (12)	0.0475 (4)	
H5	0.9074	−0.2023	0.2132	0.057*	
C6	0.94106 (15)	−0.06856 (13)	0.16406 (11)	0.0429 (4)	
H6	1.0009	−0.0894	0.1333	0.051*	
C7	0.73032 (19)	−0.18330 (17)	0.30695 (13)	0.0633 (6)	
H7A	0.6711	−0.2097	0.2750	0.095*	
H7B	0.7017	−0.1515	0.3550	0.095*	
H7C	0.7783	−0.2366	0.3228	0.095*	
C8	0.67586 (12)	0.01515 (12)	0.03229 (10)	0.0344 (3)	
C9	0.67158 (15)	0.11875 (12)	0.02304 (11)	0.0414 (4)	
H9	0.7228	0.1535	−0.0079	0.050*	
C10	0.58772 (16)	0.16563 (14)	0.06224 (12)	0.0486 (4)	
H10	0.5809	0.2344	0.0569	0.058*	
C11	0.51133 (16)	0.11404 (15)	0.11036 (12)	0.0504 (5)	
H11	0.4563	0.1499	0.1363	0.061*	
C12	0.51505 (15)	0.01289 (14)	0.12041 (11)	0.0448 (4)	
H12	0.4647	−0.0211	0.1526	0.054*	
C13	0.59940 (13)	−0.03593 (12)	0.07916 (10)	0.0349 (3)	
N1	0.71524 (13)	−0.14630 (11)	0.02730 (11)	0.0486 (4)	
N2	0.74412 (12)	−0.05667 (11)	0.00306 (10)	0.0430 (3)	
N3	0.62856 (13)	−0.13358 (11)	0.07344 (10)	0.0425 (3)	
O1	0.93251 (11)	0.11850 (10)	0.02148 (8)	0.0524 (3)	
O2	1.09402 (10)	0.08554 (10)	0.10320 (9)	0.0507 (3)	
O3	0.96735 (11)	0.21352 (9)	0.14451 (8)	0.0507 (3)	
S1	0.98089 (3)	0.11794 (3)	0.10202 (3)	0.03644 (12)	
H2A	0.8027 (13)	−0.0538 (18)	−0.0271 (13)	0.075 (8)*	
H3A	0.5961 (17)	−0.1848 (12)	0.0946 (13)	0.068 (7)*	

### Atomic displacement parameters (Å^2^)

**Table:**

	*U*^11^	*U*^22^	*U*^33^	*U*^12^	*U*^13^	*U*^23^
C1	0.0331 (8)	0.0359 (8)	0.0310 (8)	0.0049 (6)	−0.0028 (6)	−0.0038 (6)
C2	0.0387 (8)	0.0433 (9)	0.0434 (9)	0.0115 (7)	−0.0001 (7)	−0.0048 (7)
C3	0.0345 (8)	0.0616 (11)	0.0419 (9)	0.0035 (8)	0.0046 (7)	−0.0070 (9)
C4	0.0439 (9)	0.0508 (10)	0.0349 (9)	−0.0100 (8)	−0.0045 (7)	−0.0063 (7)
C5	0.0605 (12)	0.0349 (8)	0.0471 (10)	0.0004 (8)	0.0034 (9)	−0.0053 (7)
C6	0.0455 (9)	0.0392 (9)	0.0440 (9)	0.0079 (7)	0.0083 (8)	−0.0063 (7)
C7	0.0662 (14)	0.0698 (14)	0.0539 (12)	−0.0192 (11)	0.0060 (10)	0.0002 (11)
C8	0.0292 (7)	0.0383 (8)	0.0356 (8)	−0.0036 (6)	−0.0033 (6)	0.0015 (6)
C9	0.0429 (9)	0.0369 (8)	0.0443 (9)	−0.0090 (7)	0.0000 (7)	0.0052 (7)
C10	0.0580 (11)	0.0344 (8)	0.0534 (11)	−0.0022 (8)	−0.0013 (9)	−0.0022 (8)
C11	0.0490 (10)	0.0524 (11)	0.0499 (11)	0.0039 (9)	0.0062 (9)	−0.0123 (9)
C12	0.0415 (9)	0.0531 (10)	0.0399 (9)	−0.0085 (8)	0.0070 (7)	−0.0001 (8)
C13	0.0349 (8)	0.0343 (8)	0.0356 (8)	−0.0063 (6)	−0.0049 (6)	0.0023 (6)
N1	0.0438 (8)	0.0392 (8)	0.0628 (10)	0.0038 (7)	−0.0060 (8)	0.0024 (7)
N2	0.0329 (7)	0.0426 (8)	0.0536 (9)	0.0011 (6)	0.0020 (7)	0.0027 (7)
N3	0.0429 (8)	0.0352 (7)	0.0496 (9)	−0.0059 (6)	−0.0045 (7)	0.0072 (6)
O1	0.0532 (8)	0.0656 (9)	0.0383 (7)	0.0041 (7)	−0.0027 (6)	0.0063 (6)
O2	0.0346 (6)	0.0543 (7)	0.0631 (9)	0.0069 (6)	0.0037 (6)	0.0067 (6)
O3	0.0629 (8)	0.0335 (6)	0.0558 (8)	0.0072 (6)	−0.0009 (7)	0.0004 (5)
S1	0.0347 (2)	0.0360 (2)	0.0387 (2)	0.00720 (16)	−0.00031 (16)	0.00142 (16)

### Geometric parameters (Å, º)

**Table:**

C1—C2	1.383 (2)	C8—C9	1.399 (2)
C1—C6	1.385 (2)	C9—C10	1.363 (3)
C1—S1	1.7694 (17)	C9—H9	0.9300
C2—C3	1.380 (3)	C10—C11	1.404 (3)
C2—H2	0.9300	C10—H10	0.9300
C3—C4	1.386 (3)	C11—C12	1.368 (3)
C3—H3	0.9300	C11—H11	0.9300
C4—C5	1.389 (3)	C12—C13	1.395 (2)
C4—C7	1.508 (3)	C12—H12	0.9300
C5—C6	1.383 (3)	C13—N3	1.361 (2)
C5—H5	0.9300	N1—N3	1.312 (2)
C6—H6	0.9300	N1—N2	1.314 (2)
C7—H7A	0.9600	N2—H2A	0.871 (9)
C7—H7B	0.9600	N3—H3A	0.866 (9)
C7—H7C	0.9600	O1—S1	1.4423 (14)
C8—N2	1.361 (2)	O2—S1	1.4507 (13)
C8—C13	1.389 (2)	O3—S1	1.4673 (13)
			
C2—C1—C6	119.24 (16)	C10—C9—H9	122.1
C2—C1—S1	120.46 (13)	C8—C9—H9	122.1
C6—C1—S1	120.29 (13)	C9—C10—C11	122.46 (17)
C3—C2—C1	120.40 (16)	C9—C10—H10	118.8
C3—C2—H2	119.8	C11—C10—H10	118.8
C1—C2—H2	119.8	C12—C11—C10	122.29 (18)
C2—C3—C4	121.28 (16)	C12—C11—H11	118.9
C2—C3—H3	119.4	C10—C11—H11	118.9
C4—C3—H3	119.4	C11—C12—C13	115.62 (16)
C3—C4—C5	117.69 (17)	C11—C12—H12	122.2
C3—C4—C7	121.27 (18)	C13—C12—H12	122.2
C5—C4—C7	121.04 (18)	N3—C13—C8	105.09 (15)
C6—C5—C4	121.57 (17)	N3—C13—C12	132.75 (16)
C6—C5—H5	119.2	C8—C13—C12	122.16 (15)
C4—C5—H5	119.2	N3—N1—N2	105.75 (14)
C5—C6—C1	119.81 (16)	N1—N2—C8	112.14 (15)
C5—C6—H6	120.1	N1—N2—H2A	115.6 (16)
C1—C6—H6	120.1	C8—N2—H2A	132.3 (16)
C4—C7—H7A	109.5	N1—N3—C13	112.12 (14)
C4—C7—H7B	109.5	N1—N3—H3A	119.8 (16)
H7A—C7—H7B	109.5	C13—N3—H3A	128.1 (16)
C4—C7—H7C	109.5	O1—S1—O2	113.90 (8)
H7A—C7—H7C	109.5	O1—S1—O3	112.36 (8)
H7B—C7—H7C	109.5	O2—S1—O3	111.30 (8)
N2—C8—C13	104.90 (14)	O1—S1—C1	107.91 (8)
N2—C8—C9	133.48 (16)	O2—S1—C1	105.62 (7)
C13—C8—C9	121.62 (15)	O3—S1—C1	105.06 (8)
C10—C9—C8	115.83 (16)		
			
C6—C1—C2—C3	0.1 (3)	N2—C8—C13—C12	179.46 (15)
S1—C1—C2—C3	178.81 (13)	C9—C8—C13—C12	−0.8 (3)
C1—C2—C3—C4	0.9 (3)	C11—C12—C13—N3	−179.15 (18)
C2—C3—C4—C5	−0.7 (3)	C11—C12—C13—C8	1.2 (3)
C2—C3—C4—C7	179.93 (18)	N3—N1—N2—C8	−0.8 (2)
C3—C4—C5—C6	−0.5 (3)	C13—C8—N2—N1	0.63 (19)
C7—C4—C5—C6	178.86 (18)	C9—C8—N2—N1	−179.05 (18)
C4—C5—C6—C1	1.5 (3)	N2—N1—N3—C13	0.6 (2)
C2—C1—C6—C5	−1.2 (3)	C8—C13—N3—N1	−0.21 (19)
S1—C1—C6—C5	180.00 (14)	C12—C13—N3—N1	−179.87 (18)
N2—C8—C9—C10	179.18 (18)	C2—C1—S1—O1	−89.85 (15)
C13—C8—C9—C10	−0.4 (3)	C6—C1—S1—O1	88.90 (15)
C8—C9—C10—C11	1.2 (3)	C2—C1—S1—O2	147.97 (14)
C9—C10—C11—C12	−0.8 (3)	C6—C1—S1—O2	−33.28 (16)
C10—C11—C12—C13	−0.5 (3)	C2—C1—S1—O3	30.22 (15)
N2—C8—C13—N3	−0.24 (17)	C6—C1—S1—O3	−151.04 (14)
C9—C8—C13—N3	179.48 (15)		

### Hydrogen-bond geometry (Å, º)

**Table:**

*D*—H···*A*	*D*—H	H···*A*	*D*···*A*	*D*—H···*A*
N2—H2*A*···O2^i^	0.87 (1)	1.82 (1)	2.661 (2)	161 (2)
N3—H3*A*···O3^ii^	0.87 (1)	1.77 (1)	2.6326 (19)	176 (2)
C3—H3···O2^iii^	0.93	2.48	3.359 (2)	158
C12—H12···*Cg*1^iv^	0.93	2.66	3.500 (2)	150

Symmetry codes: (i) −*x*+2, −*y*, −*z*; (ii) −*x*+3/2, *y*−1/2, *z*; (iii) *x*−1/2, *y*, −*z*+1/2; (iv) *x*−3/2, *y*, −*z*−1/2.
